# Triiodothyronine prevents tissue hypoxia in experimental sepsis: potential therapeutic implications

**DOI:** 10.1186/s40635-021-00382-y

**Published:** 2021-04-09

**Authors:** Iordanis S. Mourouzis, Athanasios I. Lourbopoulos, Athanasios G. Trikas, Ioulia K. Tseti, Constantinos I. Pantos

**Affiliations:** 1 Department of Pharmacology, National and Kapodistrian University of Athens, 75 MikrasAsias Ave., Goudi, 11527 Athens, Greece; 2 Institute for Stroke and Dementia Research (ISD), University of Munich Medical Center, Munich, Germany; 3 Department of Neurointensive Care, Schoen Klinik Bad Aibling, Kolbermoorerstrasse 72 Bad Aibling, Bayern, Germany

## To the Editor,

Tissue hypoxia occurs frequently in sepsis even after apparent restoration of stable systemic hemodynamics (macro- to micro-circulation uncoupling). Low cellular oxygen content results in neo-vessel formation with abnormal vasomotor response, increased vascular permeability and thrombosis (pathologic angiogenesis) [1] and ultimately in organ failure. Thyroid hormone (TH) which is critical regulator of organ maturation, physiologic angiogenesis and mitochondrial biogenesis can adapt heart and other organs to hypoxia during development and after tissue injury later in adult life via regulation of p38 MAPK and Akt signaling pathways [2]. Along this line, the present study explored the potential of triiodothyronine (T3) to prevent tissue hypoxia which occurs early in experimental sepsis despite cardiac hemodynamics being preserved.

**Fig. 1:**
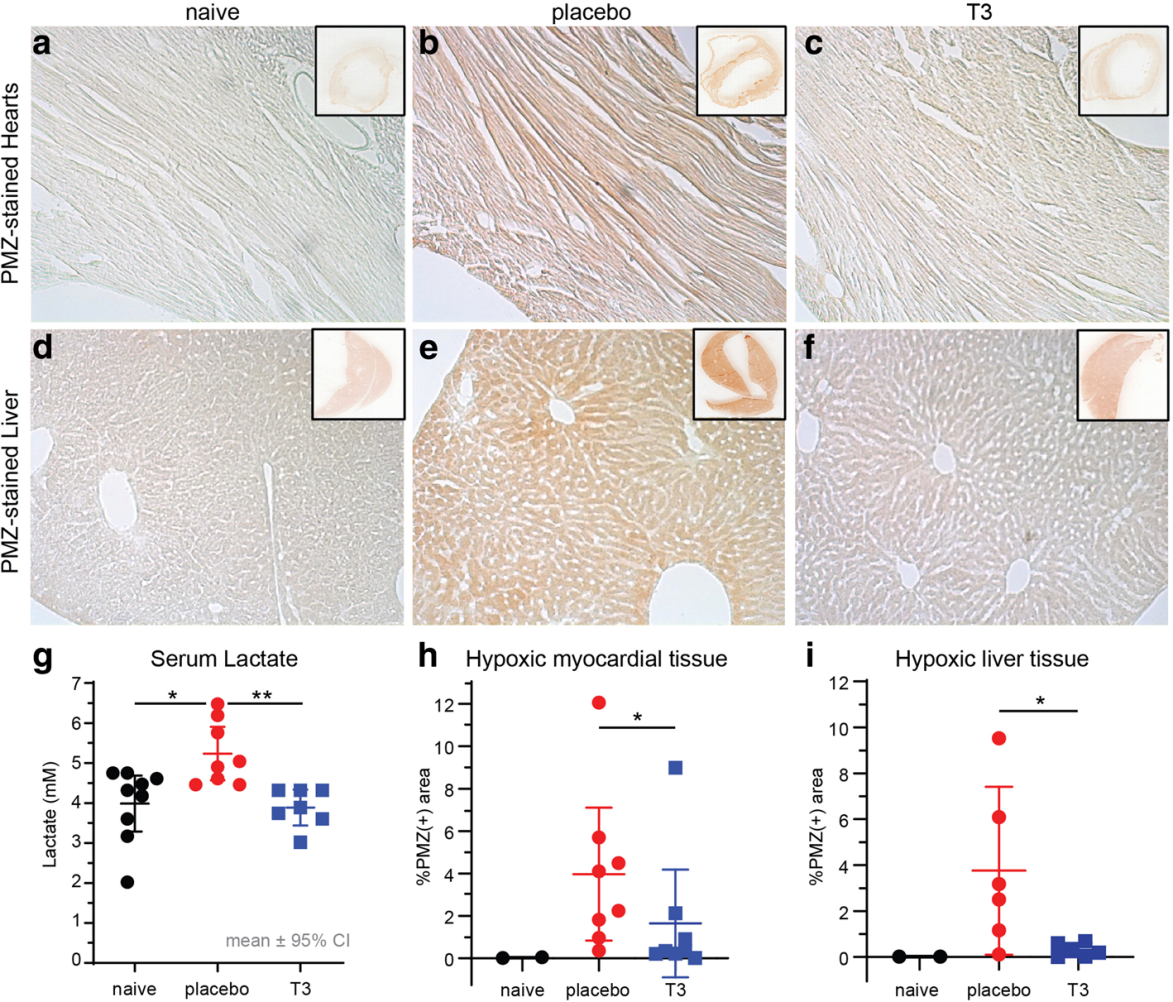
Representative microscopy images after pimonidazole (PMZ) staining from heart and liver tissues of naive (**a** and** d**), placebo (**b** and** e**) and T3-treated (**c** and** f**) animals. Detection of hypoxia (PMZ positive) was based on immunoperoxidase staining (brown color). Analysis of the pictures for detection of the PMZ-positive signal and area was performed in ImageJ using the "Threshold Color" function. Selection of threshold values was based on stained sections from naïve animals. "PMZ-positive" % area of the heart and liver tissue was identified as the percentage of pixels with color above the set threshold to total pixels in each picture. Background areas with no tissue were removed from the calculation. **g** Lactate levels in serum were 5.2 ± 0.28 mM in placebo and 4.2 ± 0.35 in T3-treated group 18 h after surgery, *p* < 0.05. Baseline lactate levels in naive animals were 4.1 ± 0.3 mM, *p* < 0.05 vs placebo only. **h** Quantification of PMZ-positive staining in left ventricular tissue was 4% ± 0.5 in placebo and 1.5% ± 0.5 in T3-treated hearts 18 h after surgery, *p* = 0.028. **i** PMZ-positive staining in liver was 3.8% ± 1.4 in placebo and 0.3% ± 0.1 in T3-treated hearts, *p* = 0.026. Results are presented as mean ± SEM. *p < 0.05 vs placebo

The protocol was approved by the Institutional Animal Care and Use Committee of Medical School, National and Kapodistrian University of Athens. Sepsis was induced in adult male 10- to 12-week-old C57BL/6 N mice by ligation distal to the ileocecal valve (25% of total cecum length) and perforation by a single 21G puncture (CLP). Animals were treated with a single dose of either vehicle (*n* = 8, placebo group) or T3 (*n* = 8, 0.3 μg/g, T3 group) intraperitoneally immediately after surgery. Naive animals were used as control (*n* = 9, naive group). Animals were killed 18 h after the CLP procedure. Lactate was measured with L-lactate assay kit in serum (Sigma-Aldrich, MAK329). Cardiac and liver hypoxia at cellular level was detected using Hypoxyprobe™ Plus kit (pimonidazole hydrochloride, PMZ) on frozen, 4% paraformaldehyde fixed tissues. PMZ was administered intravenously 2 h before the killing at a dosage of 60 mg/kg. PMZ is reductively activated in hypoxic cells (pO2 < 10 mm Hg) and forms stable adducts (sulphydryl) groups in proteins and amino acids. A specific antibody (FITC-MAb1, 1:200, overnight, 4 °C) that binds to these adducts was used combined with a chromogenic anti-FITC secondary antibody (1:200, 1 h, RT) and allowed detection by immunoperoxidase staining. Captured microscopy images were analyzed with ImageJ by automated demarcation of the PMZ-positive compared to the PMZ-negative area. Cardiac performance was assessed by echocardiography. Cardiac output (ml/min) was 14.7 (SEM, 1.0), 12.1(0.7) and 14(1.0) and heart rate (beats/min) 444(23), 439(16) and 427(9) for naïve, placebo and T3, respectively (*p* = ns). CLP resulted in increased lactate levels and cardiac and liver hypoxia at cellular level (PMZ staining) in placebo, but not in T3-treated group (Fig. 1).

TH signaling seems to be crucial in the response to lung injury in experimental sepsis and ventilator-induced trauma [3]. In addition, the present study demonstrated that T3 treatment can prevent tissue hypoxia in cardiac and liver samples which occurs early in experimental sepsis (within 18 h) before cardiac output is impaired. PMZ staining was used to detect tissue pO2 < 10 mmHg. Oxygen below this level results in activation of HIF1α-dependent regulatory mechanisms which promote pathologic angiogenesis, changes in immune response and determine sepsis-induced injury progression [4]. T3 treatment was also shown to significantly lower circulating lactate levels probably due to the prevention of tissue hypoxia. However, favorable actions of T3 on cellular metabolism may also account for this effect. T3 can improve coupling of glycolysis to glucose oxidation and decrease H^+^ production via its action on pyruvate dehydrogenase activity (PDH) [5]. PDH is found to be suppressed during sepsis [6]. This experimental evidence may be of therapeutic relevance, particularly for COVID-19 therapy where tissue hypoxia prevails [7]. Triiodothyronine has previously been administered in dopamine-dependent shock to support hemodynamics [8]. More recently, the efficacy and safety of the use of triiodothyronine has been investigated in patients with anterior STEMI undergoing angioplasty (ThyRepair trial EudraCT: 2016-000631-40). This study has been successfully completed without major safety issues [9, Suppl.Material]. Accordingly, a phase II randomized double-blind placebo-controlled study is underway to demonstrate the safety and efficacy of T3 using the same dose in critically ill COVID-19 patients (Thy-Support study, NCT04348513, EudraCT: 2020-001623-13) [9].

## Data Availability

Raw data and datasets used and/or analyzed during the current study are available from the corresponding author on request.
